# Dysregulation of the Immune Environment in the Airways During HIV Infection

**DOI:** 10.3389/fimmu.2021.707355

**Published:** 2021-06-30

**Authors:** Rubina Bunjun, Andreia P. Soares, Narjis Thawer, Tracey L. Müller, Agano Kiravu, Zekarias Ginbot, Björn Corleis, Brandon D. Murugan, Douglas S. Kwon, Florian von Groote-Bidlingmaier, Catherine Riou, Robert J. Wilkinson, Gerhard Walzl, Wendy A. Burgers

**Affiliations:** ^1^ Institute of Infectious Disease and Molecular Medicine, University of Cape Town, Cape Town, South Africa; ^2^ Division of Medical Virology, Department of Pathology, University of Cape Town, Cape Town, South Africa; ^3^ Ragon Institute of MGH, MIT and Harvard, Cambridge, MA, United States; ^4^ Institute of Immunology, Friedrich-Loeffler-Institut, Greifswald-Insel Riems, Germany; ^5^ Division of Chemical and Systems Biology, Department of Integrative Biomedical Sciences, University of Cape Town, Cape Town, South Africa; ^6^ Division of Infectious Diseases, Massachusetts General Hospital, Boston, MA, United States; ^7^ Division of Pulmonology, Faculty of Medicine and Health Sciences, Stellenbosch University, Stellenbosch, South Africa; ^8^ Wellcome Centre for Infectious Diseases Research in Africa, University of Cape Town, Cape Town, South Africa; ^9^ The Francis Crick Institute, London, United Kingdom; ^10^ Department of Infectious Disease, Imperial College London, London, United Kingdom; ^11^ DSI-NRF Centre of Excellence for Biomedical Tuberculosis Research, South African Medical Research Council Centre for Tuberculosis Research, Division of Molecular Biology and Human Genetics, Faculty of Medicine and Health Sciences, Stellenbosch University, Cape Town, South Africa

**Keywords:** lung, HIV, activation, T cells, inflammation, cytokines

## Abstract

HIV-1 increases susceptibility to pulmonary infection and disease, suggesting pathogenesis in the lung. However, the lung immune environment during HIV infection remains poorly characterized. This study examined T cell activation and the cytokine milieu in paired bronchoalveolar lavage (BAL) and blood from 36 HIV-uninfected and 32 HIV-infected participants. Concentrations of 27 cytokines were measured by Luminex, and T cells were phenotyped by flow cytometry. Blood and BAL had distinct cytokine profiles (p=0.001). In plasma, concentrations of inflammatory cytokines like IFN-γ (p=0.004) and TNF-α (p=0.004) were elevated during HIV infection, as expected. Conversely, BAL cytokine concentrations were similar in HIV-infected and uninfected individuals, despite high BAL viral loads (VL; median 48,000 copies/ml epithelial lining fluid). HIV-infected individuals had greater numbers of T cells in BAL compared to uninfected individuals (p=0.007); and BAL VL positively associated with CD4+ and CD8+ T cell numbers (p=0.006 and p=0.0002, respectively) and CXCL10 concentrations (p=0.02). BAL T cells were highly activated in HIV-infected individuals, with nearly 2-3 fold greater frequencies of CD4+CD38+ (1.8-fold; p=0.007), CD4+CD38+HLA-DR+ (1.9-fold; p=0.0006), CD8+CD38+ (2.8-fold; p=0.0006), CD8+HLA-DR+ (2-fold; p=0.022) and CD8+CD38+HLA-DR+ (3.6-fold; p<0.0001) cells compared to HIV-uninfected individuals. Overall, this study demonstrates a clear disruption of the pulmonary immune environment during HIV infection, with readily detectable virus and activated T lymphocytes, which may be driven to accumulate by local chemokines.

## Introduction

Sub-Saharan Africa has 25.6 million people currently living with HIV and 970,000 new infections a year (1). HIV-infected individuals are highly susceptible to both infectious and non-communicable pulmonary diseases such as tuberculosis [TB], *Pneumocystis* pneumonia, chronic obstructive lung disease [COPD] or pulmonary fibrosis (2–4). Although antiretroviral treatment (ART) has reduced the overall prevalence of HIV-associated lung disease, respiratory diseases still contribute to substantial morbidity and mortality in the HIV-infected population (5–7). This suggests that HIV pathogenesis extends to the lungs, requiring additional strategies to reduce the burden of respiratory diseases in HIV-infected individuals.

HIV infection is characterized by systemic immune hyperactivation and profound damage to mucosal compartments due to viral replication (8–16). Consequently, the demonstrated burden of HIV in the lung has significant implications for local pathology and impaired immunity to respiratory pathogens (17–23). HIV-associated lymphocytic alveolitis, the infiltration of lymphocytes into the airways, is associated with local viral replication (24–28). However, due to the difficulty in studying and sampling the lung compartment, the full extent of HIV-associated pulmonary immune dysfunction is not well understood.

Our previous work established that early HIV infection had a limited effect on *Mycobacterium tuberculosis* (M.tb)-specific T cell responses in BAL (29), warranting a broader investigation of the immune milieu of the lung. Therefore, in this study, we examined viral burden, T cell activation and cytokine concentrations in paired BAL and blood from HIV-uninfected and HIV-infected participants.

## Methods

### Study Participants

Participants were recruited from Cape Town, South Africa and grouped according to their HIV status: 32 ART-naive HIV-seropositive persons with CD4+ T-cell counts of >400 cells/mm^3^ and 36 HIV-seronegative persons. Participants were not eligible for this study if they had any active respiratory infections. Active TB was excluded on the basis of symptoms, radiological evidence, and BAL fluid culture results. All participants had latent TB infection (LTBI) as confirmed by a positive IFN-γ release assay (IGRA; Quantiferon-TB Gold, Qiagen, Hilden, Germany). This study was approved by the Research Ethics Committees of the University of Cape Town (REF158/2010) and Stellenbosch University (N10/08/275). All participants provided written, informed consent.

### Collection and Processing of Samples

BAL samples were collected and processed as previously described (29). Briefly, 160ml of saline was instilled in the middle lobe bronchus and aspirated. After centrifugation, acellular BAL fluid (BALF) was stored at −80°C and the cell pellet was washed and filtered through a 100-μm cell strainer (CellTrics, Partec, Münster, Germany). Cells were then counted using Trypan Blue exclusion and differentially stained in order to count macrophages, lymphocytes and neutrophils (RapidDiff, Clinical Sciences Diagnostics, Johannesburg, South Africa). The absolute number of T lymphocytes in BAL fluid was calculated using differential staining and microscopy, and the frequencies of live CD3+, CD4+, or CD8+ T cells from a flow cytometry phenotyping panel (see below). To correct for epithelial lining fluid (ELF) dilution due to variable fluid volumes recovered, the urea method was used (QuantiChrom, Clonagen, Brussels, Belgium) as described elsewhere (30). BALF viral loads and BAL cell counts were standardized according to the volume of ELF sampled (median, 1 mL; IQR, 0.75–1.64 mL) and are expressed as the number of cells or viral load per ml of ELF.

Blood specimens were collected and processed within 4 hours. Heparinized whole blood was treated with red blood cell lysis buffer without a fixative, and the cell pellet was immediately stained with a panel of antibodies for phenotyping by flow cytometry.

### Phenotyping by Multiparameter Flow Cytometry

The staining panel consisted of CCR5 PE (2D7), CD38 APC (HIT2), CD3 PE-Cy7 (SK7), HLA-DR APC-Cy7 (L243; all from BD Biosciences, New Jersey, USA), CD4 PE-Cy5.5 (S3.5), CD8 Qdot-705 (3B5), CD19 Pacific Blue (SJ25-CI), CD14 Pacific Blue (T̈k4; all from Invitrogen, California, USA), CD45RO ECD (UCHL1), CD27 PE-Cy5 (1A4CD27; both from Beckman Coulter, California, USA). Blood and BAL cells were stained with a viability marker (violet fixable viability dye, Invitrogen), followed by CCR5 labelling at 37°C before labelling with antibodies against surface markers. Cells were fixed in 1x CellFix (BD Biosciences) for acquisition a BD Fortessa using FACSDiva software. Data were analysed using FlowJo (TreeStar, Oregon, USA). Gates were set using fluorescence-minus-one (FMO) controls.

### Measurement of Soluble Analytes

A total of 27 cytokines and chemokines were measured in paired plasma and concentrated BALF samples using human magnetic bead multiplex kits (Merck Millipore, Massachusetts, USA). The Human Th17 magnetic bead kit was used to measure IL-1β, IL-4, IL-6, IL-10, IL-13, IFN-γ, GM-CSF, TNF-α, IL-21, IL-22, IL-23, IL-15, IL-17 and CCL20. The Human Cytokine/Chemokine magnetic bead kit was used to measure EGF, IL-12p70, IL-7, CXCL8 (IL-8), CXCL10 (IP-10), CCL2 (MCP-1), CCL3 (MIP-1β), CCL4 (MIP-1α), CCL5 (RANTES), CCL7 (MCP-3), CCL11 (eotaxin), CX3CL1 (fractalkine) and sCD40L. Samples were run in duplicate and the mean was calculated. Cytokine concentrations were adjusted for BAL fluid concentration factor. Cytokines that fell below the limit of detection were reported as half the minimum detectable concentration. Analytes were excluded if they fell below the empirical cut-off (either undetectable in 50% or more participants, or with a median of less than twice the minimum detectable concentration for that analyte). These were GM-CSF, IL-22, IL-4, IFN-γ, CCL11 in BAL; and GM-CSF, IL-15, IL-1β, IL-22, IL-4, IL-6 in blood. Analytes were categorized as pro-inflammatory (IL-1β, IL-6, IL-12p70, IL-23, TNF-α, sCD40L), adaptive (IFN-γ, IL-13, IL-17), γ-chain cytokines (IL-7, IL-15, IL-21), regulatory (IL-10), growth factors (EGF) and chemokines (CCL2, CCL3, CCL4, CCL5, CCL7, CCL11, CCL20, CXCL8, CXCL10, CX3CL1) based on function. The relative proportion of each analyte was calculated as a percentage of the sum total of the analyte concentrations in that compartment.

### Statistical Analyses

Non-parametric statistical analyses (Mann-Whitney U test, the Wilcoxon matched pairs test, and the Spearman rank test) were performed using Prism 7 (GraphPad). Unsupervised hierarchical clustering, principal component analyses (PCA) and permANOVA were carried out in R (31) using the following packages: pheatmap (32), vegan (33), ggfortify (34), RColourBrewer (35). False discovery rate (FDR) step down procedures were performed to adjust for multiple comparisons as previously described (36). A p value of <0.05 was considered statistically significant. The p values, p≤ 0.05, p≤ 0.01, p≤ 0.001, p≤ 0.0001 are reported as *, **, *** and ****, respectively.

## Results

### Cohort Description

Blood and BAL were collected from HIV-infected (n=32; median age, 31 years; 96% female) and uninfected (n=36; median age, 23 years; 60% female) participants from Cape Town, South Africa (**Table 1** and **Table S1**). HIV-uninfected participants had a median CD4 count of 832 cells/mm^3^ (IQR 741-1028 cells/mm^3^), while the HIV-infected individuals had a median of 601 cells/mm^3^ (IQR 523-782 cells/mm^3^; p<0.0001). HIV-infected persons were ART-naïve, however persons with CD4 counts < 400 cells/mm^3^ were excluded in order to study the impact of HIV infection prior to severe immunodeficiency. HIV-infected participants had a median HIV viral load in BAL fluid of 48,224 RNA copies/ml ELF (IQR 2,115-27,378 copies/ml ELF) and a median plasma viral load of 6,153 RNA copies/mm^3^ (IQR 2,125-17,623 copies/mm^3^; p=ns). Consistent with previous reports, there was a significant positive correlation between HIV load in BALF and plasma (p<0.0001; r=0.696; data not shown) (19, 21). These data demonstrate that despite relatively well-preserved CD4 counts, the HIV-infected group had substantial amounts of virus detectable in the airways and in blood.

**Table 1:** Clinical characteristics of study participants.

	HIV-uninfected (n = 36)	HIV-infected(n = 32)
Blood CD4 count (cells/mm^3^)	832 (741-1,028)	601 (523-782)
Plasma viral load (RNA copies/ml)	–	6,153 (2,125-17,623)
BAL viral load (RNA copies/ml ELF)	–	48,224 (2,115-27,378)

Data are median (interquartile range). BAL, bronchoalveolar lavage; ELF, epithelial lining fluid.

### Distinct Cytokine Profiles in BAL and Blood

**Figure 1:**
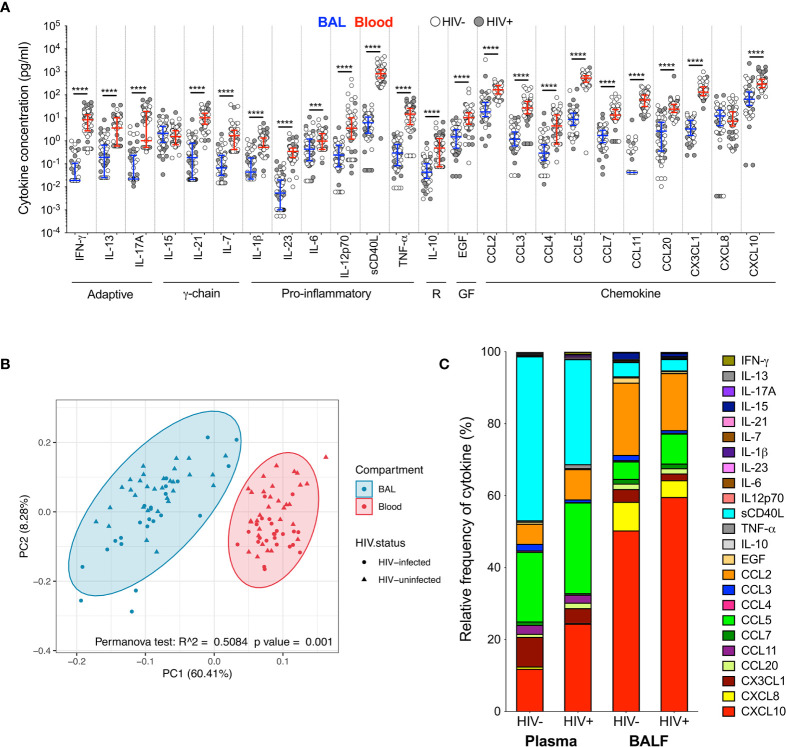
Soluble immune mediators in blood and BAL. **(A)** Comparison of cytokine concentrations in BAL (blue) and blood (red) of HIV-infected (filled circles) and HIV-uninfected (open circles) individuals. “R” refers to regulatory cytokines and “GF” refers to growth factors. The blue and red lines denote the median and interquartile ranges for BAL and blood, respectively. Statistical analyses were performed using a non-parametric Wilcoxon paired test with False Discovery Rate (FDR) step down correction. **(B)** Principal component analysis and permutational multivariate analysis of variance (permANOVA) of cytokine concentrations in BAL (blue) and blood (red). **(C)** Cytokine concentration expressed as a proportion of the total milieu in BAL and plasma. The relative proportion of each analyte was calculated as a percentage of the sum total of the analyte concentrations in that compartment. GM-CSF, IL-22 and IL-4 were excluded altogether as they were below the level of detection in both BAL and blood. Analytes that fell below the limit of detection for some participants were reported as half the minimum detectable concentration. The p values, p ≤ 0.05, p ≤ 0.01, p ≤ 0.001, p ≤ 0.0001 are reported as *, **, *** and ****, respectively.

To investigate the immune environment in the airways compared to peripheral blood, soluble cytokines and chemokines were measured in BAL fluid and blood plasma (**Tables S2, S3**). Most cytokines (22/24; 92%) were significantly higher in plasma than BAL fluid, regardless of HIV status (**Figure 1A**). Consequently, principal component analysis (PCA) demonstrated a distinct separation of cytokine profiles by compartment but not HIV status (p=0.001, r^2^ = 0.508; **Figure 1B**). We then examined the relative proportion of each cytokine adjusted to represent 100% of the overall milieu in each compartment (**Figure 1C**). Again, we observed divergent cytokine profiles between compartments. In plasma, soluble CD40L was the most abundant and made up 43% of the milieu in HIV-uninfected individuals, but only contributed 3% to the BAL cytokine profile in the same individuals. Likewise, CXCL10 contributed 44% to the milieu in BAL fluid (44%) but only 11% in plasma. Based on these observations, we focused on examining the effect of HIV infection on the airways and blood separately to account for compartmentalisation.

### The Cytokine Milieu in BAL Is Less Affected by HIV Infection Than Blood

**Figure 2:**
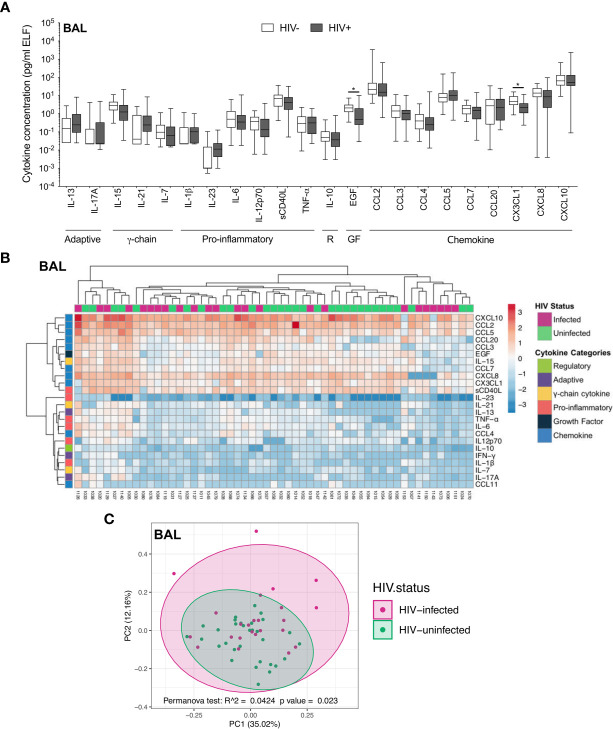
Soluble immune mediators in BAL in HIV-infected and uninfected individuals. **(A)** Box and whisker plots (min-max) comparing cytokine concentrations in BAL according to HIV status. “R” refers to regulatory cytokines and “GF” refers to growth factors. Statistical analyses were performed using a non-parametric Mann-Whitney U test with False Discovery Rate (FDR) step down correction. **(B)** Unsupervised hierarchical clustering of cytokines in BAL. **(C)** Principal component analysis and permutational multivariate analysis of variance (permANOVA) of soluble immune mediators in HIV-infected (pink; n=24) and uninfected (green; n=31) participants. GM-CSF, IL-22, IL-4, IFN-γ and CCL11 were excluded as they were below the level of detection. The p values, p ≤ 0.05, p ≤ 0.01, p ≤ 0.001, p ≤ 0.0001 are reported as *, **, *** and ****, respectively.

We first investigated the soluble cytokine milieu to elucidate which immune mediators were elevated during HIV infection. In BAL fluid, there were few differences in the soluble immune milieu between study populations. Compared to uninfected individuals, HIV-infected participants had lower concentrations of EGF (p=0.040, median: 2.02 pg/ml and 0.48 pg/ml, respectively) and CX3CL1 (p=0.044, median: 4.61 pg/ml and 2.24 pg/ml, respectively; **Figure 2A**) after correcting for multiple comparisons. Furthermore, unsupervised hierarchical clustering showed no clear clustering of cytokine profiles between HIV-infected and uninfected individuals (**Figure 2B**). Consistent with this, PCA demonstrated that cytokine profiles of the two groups did not visibly separate according to HIV status, although there was weak but significant variation in cytokine profiles between HIV-infected and uninfected groups (p=0.023, r^2^ = 0.04; **Figure 2C**).

**Figure 3:**
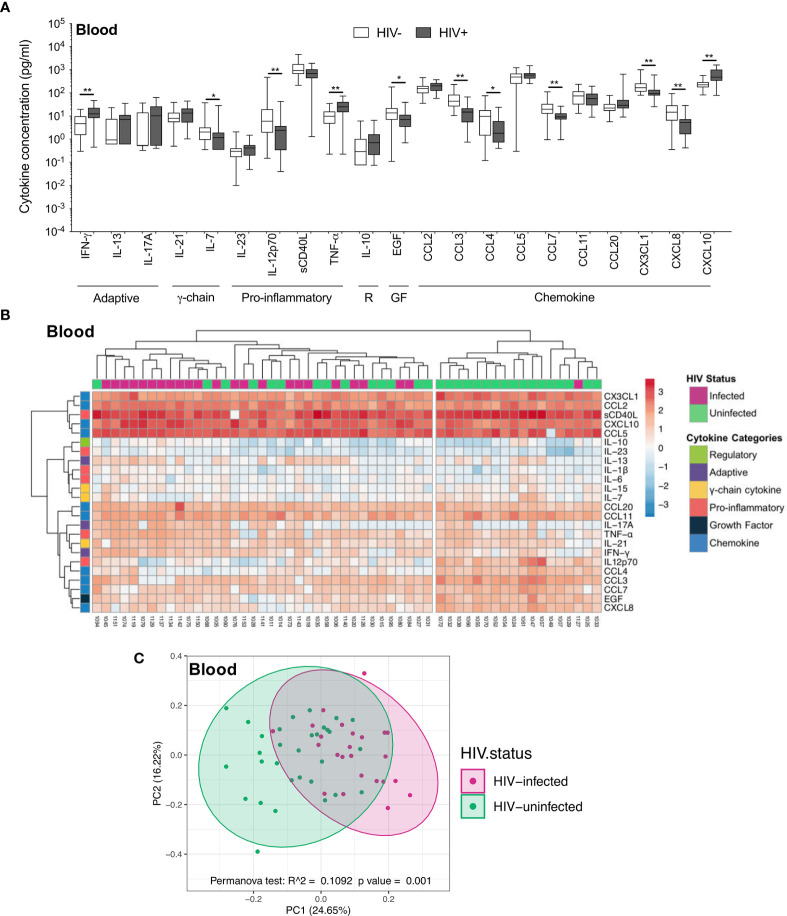
Soluble immune mediators in blood in HIV-infected and uninfected individuals. **(A)** Box and whisker plots (min-max) comparing cytokine concentrations in plasma according to HIV status. “R” refers to regulatory cytokines and “GF” refers to growth factors. Statistical analyses were performed using a non-parametric Mann-Whitney U test with False Discovery Rate (FDR) step down correction. **(B)** Unsupervised hierarchical clustering of cytokines in blood. **(C)** Principal component analysis and permutational multivariate analysis of variance (permANOVA) of soluble immune mediators in HIV-infected (pink; n=24) and uninfected (green; n=31) participants. GM-CSF, IL-15, IL-1β, IL-22, IL-4 and IL-6 were excluded as they were below the level of detection. The p values, p ≤ 0.05, p ≤ 0.01, p ≤ 0.001, p ≤ 0.0001 are reported as *, **, *** and ****, respectively.

In contrast to the airways, the plasma cytokine milieu differed considerably between HIV-infected and uninfected individuals. Compared to uninfected individuals, HIV-infected individuals had notably higher concentrations of inflammatory cytokines IFN-γ (p=0.004, median: 6.32 pg/ml *vs* 12.47 pg/ml), TNF-α (p=0.004, median: 10.18 pg/ml *vs* 25.02 pg/ml) and the chemokine CXCL10 (p=0.002, median: 219.5pg/ml *vs* 487.41 pg/ml), and lower concentrations of IL-7 (p=0.036, median: 2.21 pg/ml *vs* 1.17 pg/ml), IL-12p70 (p=0.007, median: 6.91 pg/ml *vs* 2.43 pg/ml), EGF (p=0.018, median: 15.17 pg/ml *vs* 7.07 pg/ml) and the chemokines CCL3 (p=0.002, median: 49.12 pg/ml *vs* 15 pg/ml), CCL4 (p=0.017, median: 10.4 pg/ml *vs* 1.81 pg/ml), CCL7 (p=0.002, median: 20.3 pg/ml *vs* 9.22 pg/ml), CX3CL1 (p=0.004, median: 169.7 pg/ml *vs* 95.12 pg/ml) and CXCL8 (p=0.004, median: 15.72 pg/ml *vs* 5.25 pg/ml; **Figure 3A**). Indeed, plasma cytokine profiles of HIV-infected and uninfected participants displayed a degree of clustering by unsupervised hierarchical clustering (**Figure 3B**). Similarly, principal component analysis demonstrated partial separation of cytokine profiles by HIV status *(*p=0.001, r^2^ = 0.109; **Figure 3C**). Overall, these results demonstrate that there were larger differences in the cytokine milieu between anatomical compartments than between HIV-infected and uninfected participants, with notably fewer differences observed within BAL compared to plasma, despite high BAL HIV load in these participants.

### Chemokine Concentrations Associate With T Cell Numbers and HIV Viral Load in the Airways

**Figure 4:**
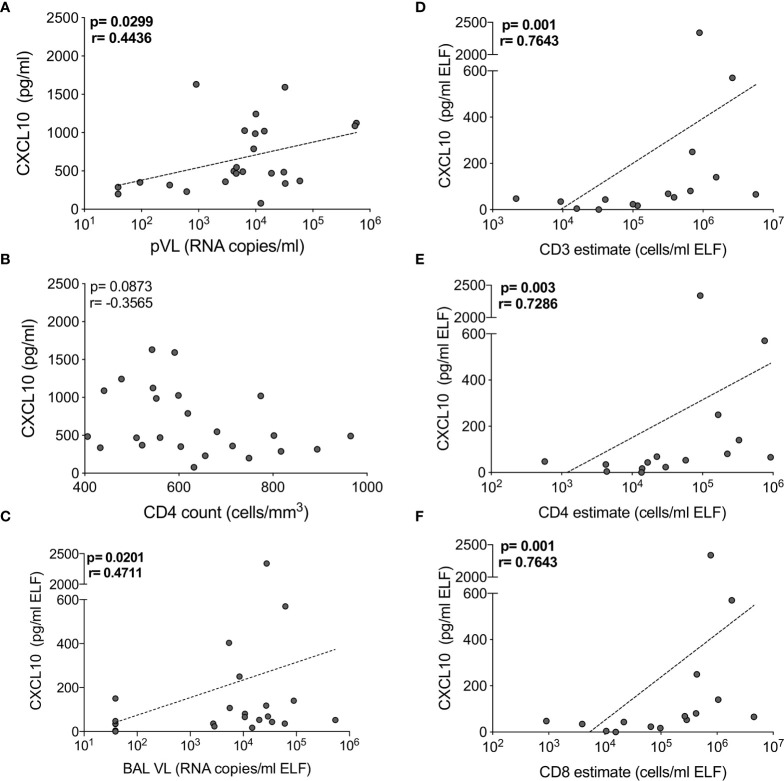
CXCL10 correlates with HIV viral load and T cell numbers in BAL of HIV-infected individuals. The correlation between CXCL10 concentration and **(A)** plasma HIV viral load, **(B)** blood CD4 count in blood, **(C)** BAL HIV viral load (n=24), **(D)** BAL CD3, **(E)** CD4 and **(F)** CD8 T cell estimates (n=16). Each dot represents an individual. Only individuals with absolute BAL cell count data were plotted. The dotted line indicates linear regression for statistically significant correlations. Statistical analyses were performed using a non-parametric Spearman rank correlation.

As reported previously (29), we found that the absolute numbers of T cells from BAL were significantly higher in HIV-infected participants, and this correlated positively with BAL viral load (**Figure S1**). To examine the interplay between HIV, the cytokine milieu and T cells, we investigated the relationships between cytokine concentrations, absolute T cell numbers and viral load. In plasma, CXCL10 concentration was significantly positively correlated with viral load (p=0.03, r=0.444; **Figure 4A**) but there was no relationship with CD4 count (p=ns; **Figure 4B**). TNF-α and sCD40L were also associated with plasma viral load (p=0.0499, r=0.405) and CD4 count (p=0.032, r=-0.439), respectively (data not shown). In BAL fluid, chemokines were significantly associated with viral load and T cell numbers. Specifically, the concentration of CXCL10 positively correlated with viral load (p=0.02, r=0.471) and the number of CD3+ (p=0.001, r=0.764), CD4+ (p=0.003, r=0.729) and CD8+ T cells (p=0.001, r=0.764) (**Figures 4C–F**). To determine whether outliers drove these correlations, we excluded the two outliers with high CXCL10 concentrations. Apart from viral load (p=0.07), the associations remained statistically significant after the exclusions. Additionally, CCL2 positively associated with BAL fluid viral load (p=0.0496, r=0.405), and CXCL8 was positively associated with numbers of CD3+ (p=0.05, r=0.516) and CD8+ (p=0.0499, r=0.516) T cells (data not shown). These associations suggest that the presence of HIV in the airways may lead to elevated levels of chemokines, and concomitant increases in T cells in the alveolar space.

### T Cells From HIV-Infected Participants Are Highly Activated in BAL and Blood

**Figure 5:**
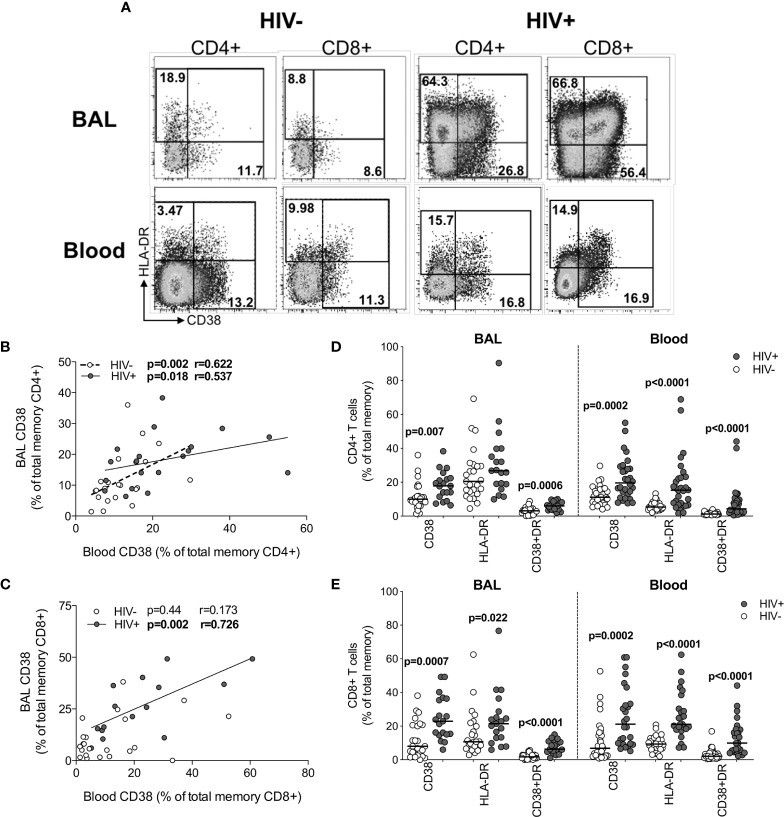
T cell activation in BAL and blood. **(A)** Representative flow cytometry plots of HLA-DR and CD38 expression on T cells in BAL and blood of HIV-infected and uninfected participants. **(B)** The association between CD4+ T cells expressing CD38 in blood and BAL of HIV-uninfected (n=22) and infected (n=15) individuals. **(C)** The association between CD8+ T cells expressing CD38 in blood and BAL of HIV-uninfected (n=22) and infected (n=15) individuals. **(D)** CD38 and HLA-DR expression on CD4+ T cells in blood and BAL of HIV-uninfected (n=31 and n=25, respectively) and infected (n=30 and n=19, respectively) individuals. **(E)** CD38 and HLA-DR expression on CD8+ T cells in blood and BAL of HIV-uninfected and infected individuals. Each dot represents an individual. Open circles represent HIV-uninfected individuals and filled circles represent HIV-infected individuals. Statistical comparisons were performed using the non-parametric Mann Whitney, Wilcoxon matched pairs and Spearman correlation tests.

Although widespread immune hyperactivation is well described during HIV infection, little is known about the activation state of lymphocytes in the airways and how this compares to peripheral blood. Thus, we characterized T cell activation, as measured by CD38 and HLA-DR expression (**Figure 5A** and **Figure S2**) and found that in HIV-uninfected individuals, frequencies of activated CD4+ T cells were higher in BAL compared to blood (for HLA-DR+ p<0.0001, median: 22.75% *vs* 5.49%; for CD38+HLA-DR+ p=0.0006, median: 3.32% *vs* 1.22%, respectively; **Figure S3A**). However, in HIV-infected individuals, there were no significant differences in CD4+ T cell activation between compartments (**Figure S3A**). There were also no differences in activated CD8+ T cells between compartments (**Figure S3B**). We observed higher frequencies of CCR5-expressing CD4+ and CD8+ T cells in BAL compared to blood, regardless of HIV status (**Figure S3C**). Furthermore, the frequencies of CD4+CD38+ and CD4+CD38+HLA-DR+ T cells between blood and BAL were positively correlated in both HIV-infected (p=0.018, r=0.537 and p=0.033, r=0.491, respectively) and uninfected individuals (p=0.002, r=0.622 and p=0.049, r=0.424, respectively**;** **Figure 5B** and data not shown). CD8+CD38+ T cells also correlated significantly between compartments, but only in HIV-infected individuals (p=0.002, r=0.762; **Figure 5C**). There was no association between T cell activation and BAL or plasma viral load (data not shown).

Direct comparison of T cell activation according to HIV status demonstrated that compared to uninfected participants, HIV-infected participants had higher frequencies of BAL CD4+ T cells expressing CD38 (p=0.007, medians 9.97% *vs* 17.8%) and co-expressing CD38 and HLA-DR (p=0.0006, medians 3.16% *vs* 6.05%; **Figure 5D**). Consistent with this, there were significantly higher frequencies of activated CD4+ T cells in blood of HIV-infected individuals compared to uninfected individuals (p=0.0002, medians 19.55% *vs* 11.1% for CD4+CD38+; p<0.0001, medians 15.25% *vs* 5.36% for CD4+HLA-DR+; p<0.0001, medians 4.29% *vs* 1.22% for CD4+CD38+HLA-DR+; **Figure 5D**). Higher CD8+ T cell activation was also demonstrated for HIV-infected individuals compared to uninfected individuals in both BAL (p=0.0007, medians 22.9% *vs* 8.0%, for CD8+CD38+; p=0.022, medians 21.4% *vs* 10.7% for CD8+HLA-DR+; p<0.0001, medians 6.3% *vs* 1.73% for CD8+CD38+HLA-DR+) and blood (p=0.0002, medians 21.15% *vs* 6.83% for CD8+CD38+; p<0.0001, medians 21.1% *vs* 9.36% for CD8+HLA-DR+; p<0.0001, medians 9.91% *vs* 2.1% for CD8+CD38+HLA-DR+; **Figure 5E**). These observations confirm that T cell activation was consistently higher in HIV-infected individuals in both BAL and blood.

### Limited Influence of BAL Cytokines on T Cell Activation

**Figure 6:**
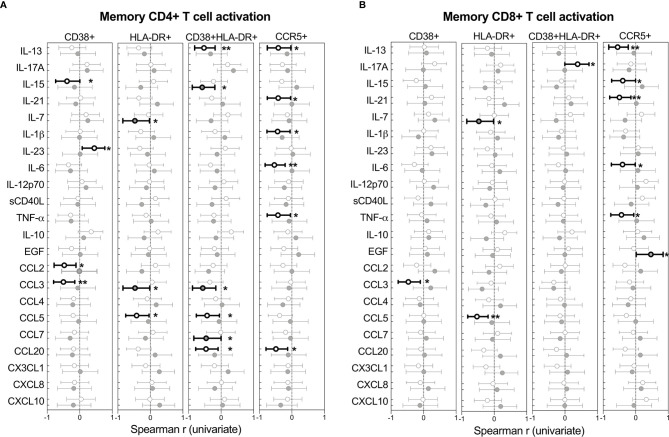
Univariate associations between T cell activation markers and cytokine concentrations in BAL of HIV-infected (n=14) and uninfected (n=21) individuals. Spearman rho (r) of the univariate correlation between each cytokine and the expression of activation markers on **(A)** CD4+ T cells and **(B)** CD8+ T cells. Open circles represent HIV-uninfected individuals and filled circles represent HIV-infected individuals. Statistically significant correlations (p<0.05) are indicated in darker lines and symbols. Spearman correlation tests, of which Spearman rho and the 95% confidence intervals are reported here. None of the correlations remained statistically significant after adjusting for multiple comparisons by FDR step down procedures. The p values, p ≤ 0.05, p ≤ 0.01, p ≤ 0.001, p ≤ 0.0001 are reported as *, **, *** and ****, respectively.

The relationships between cytokines and T cell activation in BAL was examined next. **Figure 6** shows the Spearman rho (r) of each correlation between cytokines and T cells expressing CD38, HLA-DR or CCR5. Overall, more associations between cytokines and activated T cells were observed in HIV-uninfected individuals compared to HIV-infected individuals, which could suggest some regulatory disruptions during HIV infection. However, no significant associations remained after adjusting for multiple comparisons, and linear regression analysis revealed no associations between T cell activation and cytokine concentrations (data not shown).

## Discussion

This study investigated HIV-associated immune changes in the airways, to better understand the high incidence of lung disease during HIV infection. We found distinct compartmentalisation of cytokines between BAL and blood in terms of relative cytokine abundance and cytokine concentrations, regardless of HIV status, leading us to examine the effect of HIV on each compartment individually. In BAL of HIV-infected, ART-naïve individuals, we detected a high viral load, and more T cells compared to HIV-uninfected individuals. HIV infection was also associated with increased frequencies of activated T cells. We observed a significant positive correlation between BAL viral load, absolute T cell numbers and the concentration of the chemokine CXCL10.

We detected high concentrations of HIV RNA in BAL fluid, consistent with earlier studies (19–21). The presence of HIV in the lung is likely to contribute to immunopathology and immune dysfunction, increasing susceptibility to respiratory diseases. We report a greater number of lymphocytes in HIV-infected airways, as has previously been described as lymphocytic alveolitis, thought to be predominantly made up of cytotoxic CD8+ T cells (24, 26, 27, 37). On its own, lymphocytic alveolitis causes limited pathology (25), but may contribute to the increased prevalence of pulmonary disease during HIV infection. COPD is associated with an increase in airway CD8+ T cells, particularly when combined with smoking or other risk factors (38–40). Lymphocytic alveolitis may also impair the normal response to pulmonary infections. A CD4+ T cell infiltration to the lungs would be expected in response to bacterial pathogens (41, 42), but this may be skewed towards CD8+ T cells during HIV infection. Indeed, TB-involved lung tissue from co-infected macaques (SIV and active TB) had fewer CD4+ T cells than those with active TB alone (43), suggesting SIV may interfere with the recruitment of CD4+ T cells into involved tissue. Furthermore, the infiltration of activated, cytotoxic CD8+ T cells (37), together with local pathology caused by the HIV Nef protein, may also considerably compromise mucosal barrier function via endothelial dysfunction and increased epithelial permeability (44–48). In this study, HIV-infected individuals also had lower concentrations of EGF in BAL fluid. Together, our data and these studies suggest reduced mucosal barrier function and dysregulated migration of T cells, leading to suboptimal control of infection and disease.

In HIV-uninfected individuals, BAL CD4+ T cells were significantly more activated than in blood, which is consistent with a mucosal effector environment (49–51). HIV infection led to similar levels of activated CD4+ and CD8+ T cells in BAL and blood, the likely result of systemically activated cells migrating into the airways.

We found a clear compartmentalisation of cytokine profiles between blood and BAL, with more differences between compartments than between HIV-infected and uninfected individuals. This agrees strongly with a recent study reporting distinct transcriptional profiles between BAL cells and PBMC, regardless of HIV status (37), underscoring the assertion that blood may be a poor surrogate for immune processes in the airways. In BAL, CXCL10 was present at the highest relative proportion. CXCL10 is responsible for T cell chemoattraction and is upregulated in the healthy human lung during pulmonary infection and disease (52, 53). Indeed, the preservation and increase in the BAL T cell population may be driven by the local presence of chemokines, which are elevated during HIV infection (19, 54, 55). We also found that the concentration of CXCL10 positively correlated with BAL viral load and BAL T cell numbers; and the latter two also associated with each other. These data suggest a relationship in which HIV may drive the expression of chemokines from lung cells, which in turn causes an infiltration of lymphocytes, including HIV-specific and M.tb-specific T cells (17, 18, 29, 56). We also observed elevated concentrations of the proinflammatory cytokines TNF-α and IFN-γ in plasma, consistent with previous studies (10, 57–60). In contrast, we did not detect elevated proinflammatory cytokines in BAL, despite high viral loads. Excess inflammation in the lung may cause tissue damage, which may be especially detrimental to the integrity of alveoli, so an aggressive immune response that would be permissible elsewhere is thought to be tightly controlled and regulated in the lung (61). Indeed, studies have reported that BAL CD8+ T cells have lower cytotoxic potential compared to peripheral blood CD8+ T cells (62).

Although there may be continuous migration of virus between BAL and the circulation (63), local viral replication may also be occurring. HIV target cells in the lung include small alveolar macrophages and resident CD4+ T cells expressing CCR5 (28, 64). Thus, the lung may also act as a reservoir for HIV. Previous studies have shown distinct HIV *env* sequences isolated from the lung, compared to those isolated from peripheral blood in the same individual (65, 66). Whether the lung is an important viral reservoir in the context of viral suppression and cure needs further investigation.

Our study had several limitations. We were only able to phenotype BAL T cells on a limited number of participants, due to the challenge of obtaining sufficient cells from BAL. Although BAL is representative of the bronchus, it may not necessarily reflect the immune environment of lung tissue. Further studies examining lung biopsies or other sources of lung tissue during HIV infection would give a clearer picture of HIV-associated pulmonary dysfunction. Longitudinal studies, perhaps in non-human primate models, are required to fully understand the dynamics of the immune milieu over the course of HIV infection.

In conclusion, this study demonstrates that the immune environment of the airways is disrupted during HIV infection, with readily detectable virus and the accumulation of activated T lymphocytes that may be driven by high levels of chemokines such as CXCL10 at this site. Further mechanistic studies are required to determine whether HIV-associated changes in the airways contribute to the increased susceptibility to pulmonary disease during HIV infection.

## Data Availability Statement

The raw data supporting the conclusions of this article will be made available by the authors, without undue reservation.

## Ethics Statement

The studies involving human participants were reviewed and approved by the Human Research Ethics Committee of the University of Cape Town (REF158/2010) and the Research Ethics Committee of Stellenbosch University (N10/08/275). The patients/participants provided their written informed consent to participate in this study.

## Author Contributions

WB, RB, AS, and RW conceived and designed the experiments. RB, AS, NT, TM, AK, ZG, and BC performed the experiments. RB, AS, BM, CR, and WB analyzed the data. FG-B, GW, DK, and RW contributed reagents, materials, and/or analysis tools. RB and WB wrote the manuscript. All authors contributed to the article and approved the submitted version.

## Funding

This project is part of the EDCTP2 programme supported by the European Union (EU)’s Horizon 2020 programme (TMA2016SF-1535-CaTCH-22 to WB, TMA2017SF-1951-TB-Spec to CR, TMA2020CDF-3187 to RB). Additional funding came from the Carnegie Corporation; the University of Cape Town; the Canada Africa Prevention Trials Network (all to RB); National Institutes of Health (grant R21AI115977 to CR); Wellcome Trust (203135 and 104803), NIH (U01 AI115940), the Francis Crick Institute (Cancer Research UK, MRC UK and Wellcome FC0010218), and SAMRC (SHIP) (all to RW).

## Conflict of Interest

The authors declare that the research was conducted in the absence of any commercial or financial relationships that could be construed as a potential conflict of interest.
