# Supplementary material for: Dysregulation of the Immune Environment in the Airways During HIV Infection
Source: Front Immunol. 2021 Jun 30;12:707355. doi: 10.3389/fimmu.2021.707355 (PMC8278481; doi:10.3389/fimmu.2021.707355)
Supplement: Supplementary file 1 [file DataSheet_1.docx]

| **HIV-uninfected (n=36)** | |  | **HIV-infected (n=32)** | | | |
| --- | --- | --- | --- | --- | --- | --- |
| **PID** | **CD4 count (cells/mm^3^)** |  | **PID** | **CD4 count (cells/mm^3^)** | **pVL (copies/ml)** | **BAL VL**  **(copies/ml ELF)** |
| 1001 | 631 |  | 1005 | 604 | 94 | LDL |
| 1007 | 1319 |  | 1006 | 802 | 4142 | 10746 |
| 1009 | 820 |  | 1018 | 681 | 4615 | 34941 |
| 1010 | 580 |  | 1020 | 406 | 31146 | 14660 |
| 1011 | 801 |  | 1039 | 790 | 4522 | LDL |
| 1013 | 792 |  | 1045 | 522 | 59126 | 89468 |
| 1014 | 1159 |  | 1050 | 440 | 10094 | 2255 |
| 1015 | 659 |  | 1073 | 632 | 12275 | 10701 |
| 1023 | 1120 |  | 1074 | 478 | 10094 | 61883 |
| 1024 | 939 |  | 1075 | 965 | 5923 | 5546 |
| 1025 | 915 |  | 1076 | 543 | 908 | <40 |
| 1027 | 851 |  | 1077 | 526 | 28432 | 105556 |
| 1028 | 814 |  | 1079 | 591 | 32485 | 26974 |
| 1029 | 1430 |  | 1080 | 774 | 14100 | 29094 |
| 1030 | 858 |  | 1081 | 383 | 3256 | 5324 |
| 1031 | 1169 |  | 1084 | 619 | 9192 | 2709 |
| 1032 | ND |  | 1086 | 1449 | 4250 | 7998 |
| 1033 | 813 |  | 1119 | 545 | 580150 | 544444 |
| 1035 | 1459 |  | 1126 | 433 | 32994 | 27469 |
| 1038 | 743 |  | 1127 | 817 | <40 | LDL |
| 1047 | 680 |  | 1129 | 510 | 4559 | 5394 |
| 1049 | 655 |  | 1131 | 785 | 331 | 2068 |
| 1052 | 1412 |  | 1134 | 599 | 6383 | 8449 |
| 1054 | 832 |  | 1137 | 560 | 18797 | 60444 |
| 1055 | 932 |  | 1141 | 552 | 9826 | 2850 |
| 1057 | 871 |  | 1142 | 441 | 544849 | 19992 |
| 1058 | 866 |  | 1143 | 656 | 618 | LDL |
| 1060 | 902 |  | 1150 | 894 | 311 | LDL |
| 1061 | 674 |  | 1151 | 749 | <40 | LDL |
| 1066 | 621 |  | 1152 | 571 | 9697 | 17459 |
| 1068 | 1130 |  | 1153 | 714 | 2954 | 27104 |
| 1070 | 1028 |  | 1154 | 988 | 1848 | 6023 |
| 1072 | 741 |  |  |  |  |  |
| 1094 | 827 |  |  |  |  |  |
| 1095 | 760 |  |  |  |  |  |
| 1140 | 423 |  |  |  |  |  |
| **Median** | 832 |  |  | 601 | 6153 | 48224 |
| **IQR** | 741-1028 |  |  | 523-782 | 2125-17623 | 2115-27378 |

**SUPPLEMENTARY MATERIAL**

**Table S1:** Clinical characteristics of each participant

**Table S2:** Descriptive statistics of cytokines in BAL fluid of HIV-uninfected and HIV-infected individuals.

| **Cytokines/chemokines** | **HIV-uninfected (n=31)** | |  | **HIV-infected (n=24)** | |
| --- | --- | --- | --- | --- | --- |
|  | **% Positive** | **Median (IQR)*** |  | **% Positive** | **Median (IQR)*** |
| **IL-13** | 58 | 0.14 (0.02-0.55) |  | 88 | 0.24 (0.09-0.85) |
| **IL-17A** | 39 | 0.02 (0.01-0.13) |  | 54 | 0.02 (0.02-0.28) |
| **IL-15** | 100 | 2.76 (1.58-4.44) |  | 100 | 1.26 (0.45-2.77) |
| **IL-21** | 52 | 0.03 (0.02-0.8) |  | 92 | 0.24 (0.11-0.78) |
| **IL-7** | 81 | 0.09 (0.04-0.24) |  | 75 | 0.07 (0.02-0.16) |
| **IL-1β** | 45 | 0.02 (0.01-0.17) |  | 75 | 0.1 (0.03-0.17) |
| **IL-23** | 48 | 0 (0-0.01) |  | 88 | 0.01 (0-0.02) |
| **IL-6** | 87 | 0.48 (0.2-1.37) |  | 96 | 0.35 (0.14-0.78) |
| **IL-12p70** | 87 | 0.38 (0.13-0.66) |  | 83 | 0.13 (0.06-0.51) |
| **sCD40L** | 100 | 6.59 (2.96-11.03) |  | 83 | 4.23 (1.71-7.45) |
| **TNF-α** | 87 | 0.3 (0.12-0.64) |  | 100 | 0.31 (0.08-0.61) |
| **IL-10** | 97 | 0.05 (0.03-0.1) |  | 96 | 0.04 (0.02-0.08) |
| **EGF** | 100 | 2.02 (1.3-3.15) |  | 88 | 0.48 (0.25-1.68) |
| **CCL2** | 100 | 19.9 (11.69-47.83) |  | 100 | 15.42 (9.46-44.99) |
| **CCL3** | 84 | 0.35 (0.15-0.79) |  | 88 | 0.25 (0.14-0.54) |
| **CCL4** | 94 | 1.3 (0.67-2.91) |  | 96 | 1.05 (0.47-1.53) |
| **CCL5** | 100 | 7.71 (4.86-12.84) |  | 100 | 10.43 (6.55-19.09) |
| **CCL7** | 100 | 1.83 (1.01-2.95) |  | 100 | 1.51 (0.69-2.38) |
| **CCL20** | 90 | 2.21 (0.28-6.17) |  | 100 | 2.27 (0.52-5.24) |
| **CX3CL1** | 100 | 4.61 (3.06-8.95) |  | 92 | 2.24 (1.12-3.5) |
| **CXCL8** | 97 | 13.57 (9.83-23.82) |  | 88 | 8.57 (2.15-17.88) |
| **CXCL10** | 100 | 64.69 (37.51-133.67) |  | 92 | 52.74 (34.91-123.46) |

*in pg/ml. GM-CSF, IL-22, IL-4, IFN-γ and CCL11 were excluded as they were below the level of detection.

**Table S3:** Descriptive statistics of cytokines in plasma of HIV-uninfected and HIV-infected individuals.

| **Cytokines/chemokines** | **HIV-uninfected (n=31)** | |  | **HIV-infected (n=24)** | |
| --- | --- | --- | --- | --- | --- |
|  | **% Positive** | **Median (IQR)*** |  | **% Positive** | **Median (IQR)*** |
| **IFN-γ** | 77 | 6.32 (1.7-9.85) |  | 96 | 12.47 (8.43-22.14) |
| **IL-13** | 48 | 1.95 (0.6-6.63) |  | 63 | 7.11 (0.6-11.14) |
| **IL-17A** | 39 | 0.53 (0.53-11.55) |  | 63 | 10.28 (0.53-23.72) |
| **IL-21** | 97 | 8.73 (5.64-13.34) |  | 100 | 13.55 (5.33-21.15) |
| **IL-7** | 84 | 2.21 (1.08-3.21) |  | 58 | 1.17 (0.35-1.93) |
| **IL-23** | 94 | 0.36 (0.19-0.39) |  | 100 | 0.42 (0.21-0.55) |
| **IL-12p70** | 87 | 6.91 (2.65-18.9) |  | 79 | 2.43 (0.35-3.63) |
| **sCD40L** | 100 | 1149 (682-1633.5) |  | 100 | 697.91 (468.24-1012.16) |
| **TNF-α** | 90 | 10.18 (4.85-15.35) |  | 92 | 25.02 (15.14-40.21) |
| **IL-10** | 55 | 0.27 (0.08-0.91) |  | 88 | 0.71 (0.31-1.62) |
| **EGF** | 94 | 15.17 (7.49-20.31) |  | 92 | 7.07 (3.5-10.93) |
| **CCL2** | 100 | 136.5 (103.8-199) |  | 100 | 204.15 (124.9-267.93) |
| **CCL3** | 77 | 10.4 (2.13-17.34) |  | 54 | 1.81 (0.73-5.72) |
| **CCL4** | 100 | 49.12 (26.81-79.6) |  | 79 | 15 (6.82-21.47) |
| **CCL5** | 97 | 508.13 (277.3-687) |  | 100 | 592.46 (430.15-690.72) |
| **CCL7** | 97 | 20.3 (13.01-33.12) |  | 83 | 9.22 (7.68-12.63) |
| **CCL11** | 100 | 60.4 (33.4-109) |  | 100 | 56.9 (30.48-91.17) |
| **CCL20** | 100 | 22.45 (16.99-32.8) |  | 100 | 29.6 (21.68-48.94) |
| **CX3CL1** | 100 | 169.7 (116.6-256) |  | 100 | 95.12 (78.14-133.66) |
| **CXCL8** | 100 | 15.72 (6.48-28.46) |  | 100 | 5.25 (2.07-7.2) |
| **CXCL10** | 100 | 219.5 (176.4-287) |  | 100 | 487.41 (346.46-1021.42) |

*in pg/ml. GM-CSF, IL-15, IL-1β, IL-22, IL-4 and IL-6 were excluded from analysis as they were below the level of detection

**Figure S1:** **Estimated absolute numbers of CD3+, CD4+ and CD8+ T cells in BAL.** (A) The absolute number of CD3+, CD4+ and CD8+ cells in BAL of HIV-infected (n=23) and uninfected (n=25) individuals. Data are shown as box and whisker (interquartile range) plots and horizontal bars represent the median. White and grey bars represent HIV-uninfected and infected individuals, respectively. (B) The association between CD4+ and CD8+ numbers and plasma viral load. Each dot represents an individual. Open squares represent HIV-uninfected individuals and filled squares represent HIV-infected individuals. Statistical comparisons were performed using a non-parametric Mann Whitney test and non-parametric Spearman rank correlation.

**Figure S2: Gating strategy to identify activated T cells from (A) BAL and (B) whole blood.** Populations shown are after time gates to exclude uneven fluorescent signal during acquisition. Gates were defined based on fluorescence-minus-one (FMO) controls. These representative samples are from HIV-uninfected individuals.

**Figure S3: Comparison of the frequencies of activated T cells between blood and BAL.** (A) Comparison of the frequency of CD4+ T cells expressing CD38, HLA-DR or both in matched blood (red) and BAL (blue) of HIV-uninfected (n=22) and infected (n=15) individuals. (B) Comparison of the frequency of CD8+ T cells expressing CD38, HLA-DR or both in matched blood (red) and BAL (blue) of HIV-uninfected (n=22) and infected (n=15) individuals. (C) Comparison CD4+ and CD8+ expressing CCR5 in matched blood (red) and BAL (blue) of HIV-uninfected (n=22) and infected (n=15) individuals. Statistical comparisons were performed using the non-parametric Wilcoxon matched pairs tests.
